# Cases from Dr. Mott’s Clinique, University of City of New York

**Published:** 1847-05

**Authors:** 


### *Cases from* Dr. Mott's *Clinique, University of City of New York.*

*Reported for the New York Annalist.*

*Facial Neuralgia.*—A man aged 34, complaining of flashes of pain, and twitches affecting the side of the face, so severe as to make his eyes water. They were brought on almost invariably by spitting, blowing the nose, sneezing, washing the face, or entering a warm room. The latter circumstance, Dr. M. said, was common in cases in which exposure to the cold would relieve the suffering. The pain, in the patient, had commenced about a year ago, in the infra-orbitary region, and was confined chiefly to the cheek and nostril, which were puffy and tender to the touch. Now, it had extended itself, embracing the whole side of the face. It was, said Dr. M., a well marked example of Tic Doloureux, or Facial Neuralgia, commencing as it usually did, in the 2d branch of the fifth pair, and afterwards involving, in its course, the first and third branches. The general health of the patient was good, and the cause, as was often the case, not evident. Treatment, in these cases, was too often unavailing. The strong Tincture of Aconite, prepared according to Fleming's direction, in doses of four or five drops, three times a-day, and increased by a drop at a dose every two days, is one of the best remedies. It should also be applied locally. He had also often found stramonium, which is one of the best narcotics (anodynes) which we possess, extremely suitable in these cases.

*Struma.*—A boy about ten years old. This child, said Dr. M., interests me. He is strumous *a capite ad calcem.* There is trouble, probably of that kind, in the testis. If so, it is the earliest case of tuberculous testis I have ever seen. It is rare to see either strumous, or malignant disease affect that organ before its function is developed. A case of malignant cerebriform disease of the testis, in a child 8 years old, is related by the younger Cline, in the Med. Ch. Transact.; but that is *sine pare*, as far as I know. There is also a well-authenticated case on record, of congenital malignant disease in the testis. These are among the white-black-birds of the day. If any disease does occur in this case, it will be strumous. It has not the appearance of malignant disease. I mean a disease which spreads by absorption. I hope it will continue scrotal, but I fear it may be otherwise. The testis is very tender, and threatens to become involved. He has also a cold abscess in his back, which has been opened, but is now again fluctuating. It is evidently a case of whole Struma, and is *blowing out*, (where it is rare for it to do so) in the scrotum. This serves as a vent-hole for the disease. This "*blow-out*," is a good thing in some respects. Sir A. Cooper used to say, when he had the gout, that he was very glad of it.—It blew out there, and after that, he was always a great deal better. The same language is held by other gouty old gentlemen.—Sir. A. Cooper was a good English feeder, and had, from his profession, a revenue only surpassed by that of a distinguished lawyer, Sir Samuel Romilly. His was £21,000, or £22,000, sterling per annum; the latter's £31,000, or £32,000. Think of that, for comfort. But this is rare. If a child has lymphatic glandular struma, and you do not do too much, and keep it there, it is very well.—If you discuss them, the disease goes to the lungs, and tuberculization ensues. When I see an adult with enlargement of the lymphatic glands of the neck, who is predisposed to phthisis, I am very careful how I try to remove them. Much more is done by conducting a disease along, than by forcing. Doctors don't cure fevers; and more are cured by the French system of syrup of gum, and *ptisanes*, than by stiff treatment. The great business of the physician, in fever, is to conduct it along; it is like the sailing of a ship. I do not of course apply these remarks to miasmatic fevers. This boy is taking an alterative and tonic, composed of Corr. Sub. in Tinct. Cinchon. We will go on with it for a little while. I fear for the consequences of the disease in that organ below. The patient was then ordered a suspensory bag, the testis to be rubbed (experimentally), with an Ung. Iodur. Plumb., and covered with oiled silk. To return in two weeks. The Professor repeated his assertion, that struma cannot be cured by depletion.

*Struma.*—This boy had been several times before at the clinique. Dr. M. remarked, that he was entirely strumous. He had disease of the testis, of the back, of the femur, and seems now to be getting hip joint disease. A seton should be inserted behind the great trochanter, or in the fold of the groin, as that is nearest to the joint. You observe, in this case, that there has been diversion from the hip joint by the disease of the testis. We often have occasion to observe a discharge from some other point to relieve the organ affected with strumous disease. On this principle, and knowing by experience the value of the seton, we prescribe it in this case.—Yesterday I saw a little boy with osteitis of the tibia; he had formerly disease of the knee joint, which diminished as the disease of the tibia increased, and a discharge took place.

I have long been convinced of the value of issues in affections of the knee joint. They have done more good than all the iodides in creation. They should be applied below the joint, and on the inside. I do not like applications, blisters, &c., *over* the joint.—This boy should have good food, meat, &c., and have daily some beer, or good wine, if that could be obtained. This disease (struma,) is exceedingly common in Paris, where the number of poor is very great, and they are crowded together in streets where the sun scarcely reaches them during the day. I have frequently heard Guerin prescribe for these scrofulous patients—meat diet, the red wine of the country, and exercise *in the open light of the sun.* He has tried many experiments on dogs, by keeping them in damp cellars, deprived of light, and found that struma was invariably produced in every instance.—*Med. and Surg. Reporter.*

